# Haptic Amputation Under Endoscopic Guidance in Uveitis-Glaucoma-Hyphema Syndrome: A Case Report

**DOI:** 10.7759/cureus.36303

**Published:** 2023-03-17

**Authors:** Merai Alshehri, Ali Al Beshri, Dania Bamefleh

**Affiliations:** 1 Glaucoma Department, King Khaled Eye Specialist Hospital, Riyadh, SAU; 2 Department of Surgery, College of Medicine, University of Bisha, Bisha, SAU

**Keywords:** uveitis-glaucoma-hyphema syndrome, intraocular implants, haptic amputation, intraocular pressure, endoscopic cyclophotocoagulation, uveitis glaucoma hyphema syndrome

## Abstract

Uveitis-glaucoma-hyphema (UGH) syndrome is a rare ophthalmic postoperative complication in which the intraocular implants or devices like intraocular lenses (IOLs) produce chronic mechanical chaffing to the adjacent uveal tissues and/or trabecular meshwork (TM) resulting in a wide spectrum of clinical ophthalmic manifestations ranging from chronic uveitis to secondary pigment dispersion, iris defects, hyphema, macular oedema, or spiked intraocular pressure (IOP).

Spiked IOP is a result of direct damage to the TM, hyphema, pigment dispersion, or recurrent intraocular inflammation. UGH syndrome generally develops over a time course, varying from weeks to several years postoperatively.

Conservative treatment with anti-inflammatory and ocular hypotensive agents might be sufficient in mild to moderate UGH cases but surgical intervention with implant repositioning, exchange, or explantation might be necessary in more advanced situations.

Here, we report our challenge in managing a one-eyed 79-year-old male patient with UGH secondary to migrated haptic, which was successfully managed by intraoperative IOL haptic amputation under endoscopic guidance.

## Introduction

Uveitis-glaucoma-hyphema (UGH) syndrome was first coined by Ellingson in 1978 and it is a rare postoperative complication in which the intraocular implants or devices with irregular placement or inappropriate design (e.g. intraocular lenses (IOLs)) produce chronic mechanical chaffing to the adjacent uveal tissues (iris or ciliary body) and/or trabecular meshwork (TM) resulting in a wide spectrum of clinical manifestations ranging from chronic uveitis to secondary pigment dispersion, iris transillumination defects (TIDs), hyphema, macular oedema, or spiked intraocular pressure (IOP) [1-4].

Direct damage to TM, hyphema, pigment dispersion, or recurrent inflammation all contribute to repeated elevated IOP attacks, which may result in glaucomatous optic neuropathy [3,4].

UGH syndrome generally develops over a time course, varying from weeks to several years postoperatively [5-7].

The management of UGH syndrome involves a step ladder approach corresponding to the severity. Conservative treatment with anti-inflammatory and ocular hypotensive agents might be sufficient in mild to moderate cases but surgical intervention with implant repositioning, exchange, or explantation might be necessary in more advanced situations [4,5,7,8].

In this article, we describe a unique case of UGH secondary to migrated haptic, which was successfully managed by intraoperative IOL haptic amputation under endoscopic guidance.

## Case presentation

A 79-year-old male presented to the emergency department of our hospital with complaints of the right eye (seeing eye) mild to moderate ocular pain associated with a gradual decrease of vision over two years, which started getting worse in the last two months. These complaints were not associated with headache, nausea, or vomiting. His medical history was notable for hypertension on medications, including acetylsalicylic acid (aspirin) 81 mg once daily and valsartan (Diovan) 80 mg once daily.

His past ocular history included uneventful phacoemulsification with IOL implantation eight years ago in both eyes at a private centre. Corneal decompensation followed the cataract surgery in the left eye, and then the patient underwent two corneal graft surgeries that failed in the end. Since then, the right eye is considered the good-seeing eye. There was no history of previous ocular trauma, pre-existing glaucoma, or chronic use of ocular drops.

On initial examination, his uncorrected visual acuity (VA) was hand motion (HM) in both eyes with no improvement with pinhole. The IOP measured with the Goldmann applanation tonometer (GAT) was 39 mmHg and 08 mmHg in his right and left eye, respectively.

**Figure 1:**
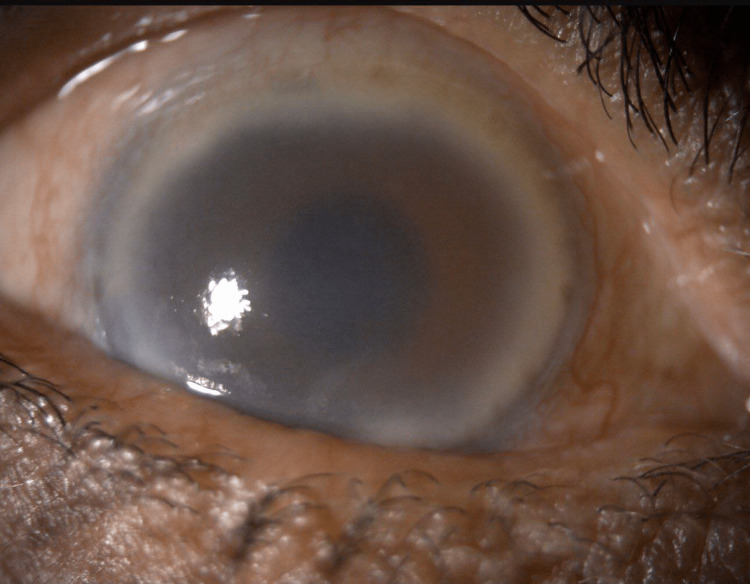
Slit lamp photo of the right eye at presentation

Slit lamp examination for the right eye (Figure 1) showed mildly injected conjunctiva and moderately diffuse corneal oedema with signs of anterior uveitis, including 2+ cells, flare, and pigments with 1 mm height of hyphema with no further view behind.

**Figure 2:**
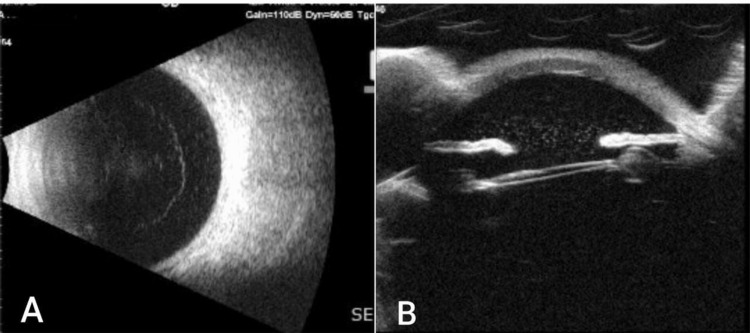
B-scan of the right eye showing flat retina with no vitreous haemorrhage (A). UBM of the same eye showing subluxated IOL touching the iris (B) UBM: ultrasound biomicroscopy; IOL: intraocular lens.

The grafted cornea in the left was diffusely oedematous with scarring with no view behind. B-scan ultrasonography was unremarkable for both eyes. Ultrasound biomicroscopy (UBM) of the right eye revealed a subluxated IOL inferiorly touching the iris (Figure 2).

The patient was diagnosed with right UGH syndrome secondary to a subluxated IOL. The patient was admitted to the ward and started on topical atropine sulphate 1% three times a day, prednisolone acetate 1% every four hours, and full anti-glaucoma drops, including brimonidine 0.2%, timolol maleate 0.5%, brinzolamide 1%, and bimatoprost 0.01%.

On the first day of admission, the patient noticed that ocular pain was less in severity but with a similar clinical picture and the IOP was still high at 34 mmHg. We decided to increase the frequency of steroid drops to every two hours and to add acetazolamide 250 mg tablet (Diamox) twice daily after getting medical clearance.

On the second day of admission, the patient symptomatically improved; he had less pain and his vision become more clear, with a VA of 20/400. His IOP went down to 28 mmHg and a slit lamp exam revealed less oedematous cornea, an otherwise similar finding to the day of admission. We decided to continue the same treatment plan with limited physical activity and maintaining head elevation during sleep.

On the third day of admission, the patient's vision deteriorated to HM despite a normal IOP of 18 mmHg and hyphema height increased and the cornea became oedematous again but with no corneal staining noticed. We then decided to continue the same treatment plan and to prepare the patient for surgery (anterior chamber (AC) wash out +/- IOL repositioning, exchange, or removal) the next day after discussing with him all benefits, risks, and alternatives of this intervention.

Surgical technique

**Figure 3:**
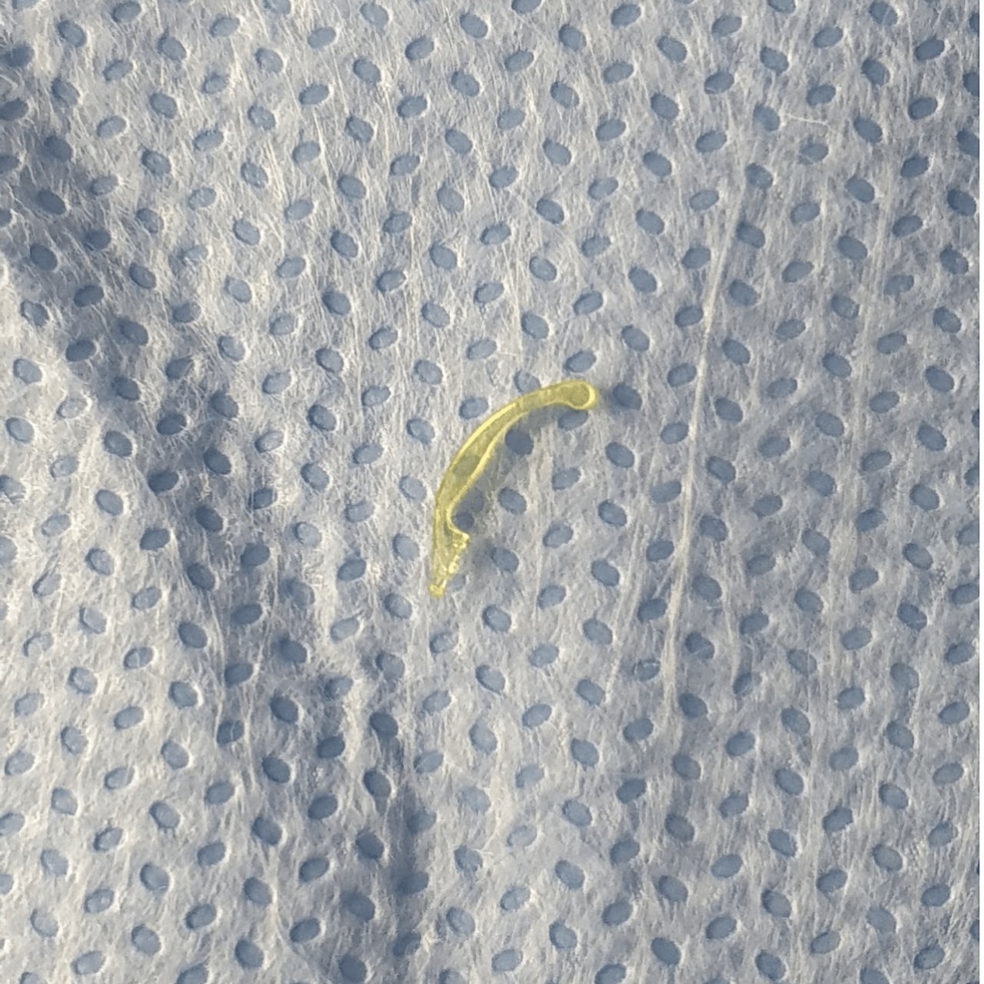
Amputated intraocular lens haptic

A temporal and nasal paracentesis was made using a microvitreoretinal (MVR) 23 blade (Alcon Laboratories, Inc., Fort Worth, TX). We injected preservative-free Mydrane (a combination of tropicamide 0.02%, phenylephrine 0.31%, and lidocaine 1%) intracamerally. A main wound was created temporally using keratome 2.2 mm (Alcon Laboratories, Inc.). We injected an ophthalmic viscosurgical device (OVD) (dispersive viscoelastic) (Alcon Laboratories, Inc.) to maintain the AC, wash out the blood, and dilate the pupil. Using an endoscopic cyclophotocoagulation (ECP) probe (Endo Optiks, Little Silver, NJ), we went in and saw migrated haptic at the sulcus while the other one was in the bag. Extensive fibrosis was noticed. We amputated the anterior haptic using micro-scissors (Synergetics, B+L, St. Louis, MO) and it was delivered out with McPherson forceps (Figure 3).

We performed endolaser treatment at 2.5 watts power to 270 degrees of ciliary body processes with a fair response. We washed OVD and closed the wounds with stromal hydration and the main wound using 10-0 nylon sutures.

**Figure 4:**
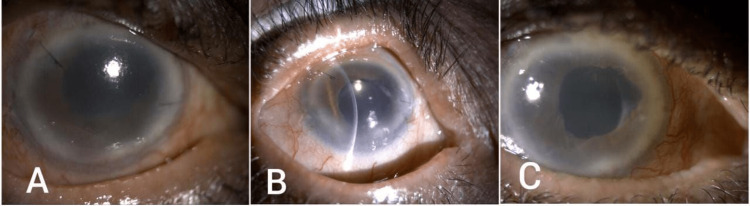
Slit lamp photo of the right eye on day one (A), four weeks (B), and six months (C) postoperatively

On the first postoperative day (Figure 4A), the uncorrected VA in his right eye was counting fingers at one metre and the IOP was 20 mmHg on Diamox twice a day (BID). A slit lamp examination showed less oedematous cornea with 1+ cells in the AC without hyphema, and the IOL was stable and well-centred in the bag. The patient was discharged home on atropine, steroid, timolol, and brimonidine drops in addition to Diamox tablet 250 mg BID. Two weeks postoperatively, the uncorrected VA was 20/200 with an IOP of 19, and the cornea became more clear, with a quiet AC and IOL in place. One month postoperatively (Figure 4B), the uncorrected VA was 20/70 with IOP of 11 mmHg, clear cornea, quiet AC, and IOL in place with good red reflex, and the patient was advised to start tapering steroid drops, stopping Diamox tablet, and continuing topical anti-glaucoma drops with timolol and brimonidine. On his last examination, six months postoperatively (Figure 4C), the uncorrected VA was 20/40, the IOP was 14 mmHg, and a slit lamp exam showed clear cornea, quiet AC, well-positioned IOL, and flat retina with 0.75 cupping.

## Discussion

UGH syndrome or Ellingson syndrome was first coined by Ellingson in 1978 [1]. It is a rare postoperative complication in which the intraocular implants or devices, mainly IOLs (either anterior or posterior IOLs) or iris prostheses, produce chronic mechanical chaffing to the adjacent uveal tissues (iris and/or ciliary body) and/or TM resulting in a wide spectrum of clinical findings including elevated IOP [1,3]. It is less often seen with devices such as glaucoma implants (e.g. Ahmed implant, Ex-Press shunts, iStent, and Hydrus Microstent).

Direct damage to TM, hyphema, pigment dispersion, and recurrent inflammation contribute to repeated elevated IOP attacks, which may result in glaucomatous optic neuropathy [4,5].

Factors such as irregular implant placement, such as IOLs implanted in the anterior chamber, inappropriate implant design, such as square-edged IOL, or improper surgical technique, like implanting single-piece IOL in the sulcus, contribute to the incidence of UGH syndrome [6,7].

UGH syndrome has sharply declined due to advancements in lens types, designs, surgical techniques, and the use of posterior chamber IOLs [4].

The diagnosis of UGH is clinical, based on patient history and slit lamp exam, and is supported by imaging. The patients often present with blurry vision, intermittent ocular pain, redness, and photophobia, and all these symptoms fluctuate and correspond to the severity of the case [7].

UGH syndrome has a wide spectrum of clinical manifestations ranging from chronic uveitis to pigment dispersion, recurrent hyphema, cystoid macular oedema, elevated IOP, iris TIDs or neovascularization with malpositioned IOL, or haptic, as in our case [9-11].

UBM is a useful tool to aid in the diagnosis of UGH by visualization of IOL position and to comment on any contact with the iris or ciliary body, especially in those cases with difficulty seeing the IOL by slit lamp exam due to corneal oedema or hyphema, as in our case [12].

B-scan is also a helpful tool to rule out posterior segment pathology in cases with no view to the fundus [4].

UGH syndrome generally develops over a time course, varying from weeks to several years postoperatively and its management involves an approach corresponding to the severity, frequency of the signs and symptoms, lens type, position, and duration since primary surgery was done [6-8].

Topical treatment with anti-inflammatory and/or ocular hypotensive in addition to close observation might be enough in addressing mild to moderate cases, while surgical intervention is mandatory in advanced cases with longstanding inflammation, hyphema, and persistently high IOP [4,7,9,13].

In cases of IOL AC or sulcus (with single-piece lenses) implantation, UGH is more frequently encountered. When the patient starts to be symptomatic by signs and symptoms of UGH, it is recommended to either reposition the IOL or replace it with a more suitable IOL type or design (three-piece IOL) in a better position [11].

The optic of the IOL is in the capsular bag, but, occasionally, one of the haptics is unintentionally placed in the sulcus, or it migrates to the sulcus with progressive postoperative capsular fibrosis leading to UGH, as in our case [9].

In the early postoperative period, this can be managed simply by reinserting the haptic in the capsular bag after inflating the bag with OVD. Capsular fibrosis with extensive adhesion precludes haptic repositioning in longstanding cases making either IOL removal or exchange the only solution [7,9].

Due to the risk of capsular rupture, uveal tissues injury, corneal endothelial dysfunction, or dislocated capsular bag, especially in the absence of clear visualization of the AC, iris, and IOL, we found that simply amputating or cutting the migrated haptic at its junction with optic under endoscopic guidance by ECP probe would be the best choice with less time-consuming, less traumatic, and encouraging outcome.

## Conclusions

UGH is a rare ophthalmic postoperative condition with a wide spectrum of clinical manifestations that develop over a time course, varying from weeks to several years postoperatively and ranging from chronic uveitis to secondary pigment dispersion, iris TIDs, hyphema, macular oedema, or uncontrolled IOP.

The management of UGH syndrome involves a step ladder approach corresponding to the severity. Conservative treatment might be sufficient in mild to moderate cases but surgical intervention might be necessary in more advanced situations. In cases of UGH induced by migrated haptic in the sulcus with no view of the implanted IOLs perioperatively, it can be managed safely and successfully by intraoperative IOL haptic amputation under endoscopic guidance.
